# Stimulation of primary osteoblasts with ATP induces transient vinculin clustering at sites of high intracellular traction force

**DOI:** 10.1007/s10735-013-9530-7

**Published:** 2013-08-10

**Authors:** Toh Weng Tan, Bastian Pfau, David Jones, Thomas Meyer

**Affiliations:** 1 Institute for Experimental Orthopaedics and Biomechanics, University of Marburg, Marburg, Germany; 2 Institute for Optics and Atomic Physics, Technical University of Berlin, Berlin, Germany; 3 Department of Psychosomatic Medicine and Psychotherapy, University of Göttingen, Waldweg 33, 37073 Göttingen, Germany

**Keywords:** Purinergic stimulation, Extracellular ATP, Vinculin, Cytoskeleton, Osteoblasts

## Abstract

Adenosine 5′-triphosphate (ATP), released in response to mechanical and inflammatory stimuli, induces the dynamic and asynchronous protrusion and subsequent retraction of local membrane structures in osteoblasts. The molecular mechanisms involved in the ligand-stimulated herniation of the plasma membrane are largely unknown, which prompted us to investigate whether the focal-adhesion protein vinculin is engaged in the cytoskeletal alterations that underlie the ATP-induced membrane blebbing. Using time-lapse fluorescence microscopy of primary bovine osteoblast-like cells expressing green fluorescent protein-tagged vinculin, we found that stimulation of cells with 100 μM ATP resulted in the transient and rapid clustering of recombinant vinculin in the cell periphery, starting approximately 100 s after addition of the nucleotide. The ephemeral nature of the vinculin clusters was made evident by the brevity of their mean assembly and disassembly times (66.7 ± 13.3 s and 99.0 ± 6.6 s, respectively). Traction force vector maps demonstrated that the vinculin-rich clusters were localized predominantly at sites of high traction force. Intracellular calcium measurements showed that the ligand-induced increase in [Ca^2+^]_i_ clearly preceded the clustering of vinculin, since [Ca^2+^]_i_ levels returned to normal within 30 s of exposure to ATP, indicating that intracellular calcium transients trigger a cascade of signalling events that ultimately result in the incorporation of vinculin into membrane-associated focal aggregates.

## Introduction

In addition to its well-known role as an intracellular energy store, adenosine 5′-triphosphate (ATP) functions as a signalling molecule in the extracellular environment, where it regulates inflammatory responses, synaptic neurotransmission, and remodelling of bone architecture. Irrespective of whether this nucleotide is inside or outside the cell, ATP is indispensable for numerous signal transduction cascades and mediates a variety of cellular behaviour. Inside the cell, ATP is generated by glycolysis, citric acid cycle, β-oxidation or oxidative phosphorylation, and is engaged in myosin-dependent contractions, F-actin polymerization, cell motility and biosynthetic pathways. There is growing evidence that extracellular ATP is an important modulator for a wide range of intercellular communications, including hormone action, wound healing, salivary secretion, and the formation of inflammasome signalling complexes that promote the release of cytokines (Klepeis et al. 2004; Ihara et al. 2005; Kaplan et al. 1995; Kudirka et al. 2007; Taboubi et al. 2007; Dubyak 2012).

In osteoblasts, stimulation with ATP activates DNA synthesis and enhances the proliferative effects of growth factors via a mitogen-activated protein kinase pathway (Nakamura et al. 2000; Katz et al. 2006, 2008). Extracellular ATP exerts osteolytic effects on bone structure both by enhancing the bone-resorbing activity of osteoclasts and by suppressing the mineralization of collagenous matrix by osteoblasts (Bowler et al. 2001; Hoebertz et al. 2002; Buckley et al. 2002; Orriss et al. 2007). Hypoxia has been shown to enhance vesicular ATP release in osteoblasts, suggesting that local purinergic signalling contributes to the detrimental effects of reduced O_2_ tension on bone cells at sites of tissue damage, bone fracture or inflammation (Orriss et al. 2009, 2010).

Nucleotides such as ATP and uridine triphosphate (UTP) signal through purinergic P2 receptors which are subdivided into G-protein-coupled P2Y receptors and P2X ligand-gated ion channels (Gallagher and Buckley 2002; Gartland et al. 2012). Activation of P2 receptors in bone cells by extracellular ATP leads to an increase in the intracellular Ca^2+^ concentration (Jørgensen et al. 1997; Katz et al. 2006, 2008; Liu et al. 2008; Nishii et al. 2009; Grol et al. 2012). It has been shown that ATP stimulation via P2X_7_ receptors mediates skeletal mechanotransduction and is necessary for mechanically induced release of prostaglandins by bone cells (Li et al. 2005). Additionally, it has been reported that mechanical stretching of osteoblastic cells results in the secretion of ATP which can partially be blocked by the L-type voltage sensitive calcium inhibitor nifedipine (Romanello et al. 2001; Hecht et al. 2013). Exposure of bone-derived cells to high concentrations of ATP leads to a dramatic and reversible increase in membrane blebbing (Verhoef et al. 2003; Pfeiffer et al. 2004; Panupinthu et al. 2007, 2008; Hwang et al. 2009). Ligand-induced membrane blebbing is associated with dynamic and reversible binding of calmodulin to the carboxy-terminus of P2X_7_ receptors and induces a series of cytoskeletal and mitochondrial alterations (Roger et al. 2008). The ATP-induced cell blebs appear as local herniations of the plasma membrane and can be transformed from protrusions with typical spherical shape into lamellipodia and vice versa, a process commonly termed zeiosis (Godman et al. 1975). Membrane blebbing is observed upon drug treatment and induction of apoptotic cell death, but it is also engaged in physiological processes such as mitosis and ligand-induced receptor binding (Baldini et al. 2008; Silber et al. 2012).

In this study, we report on the transient redistribution of the focal-adhesion protein vinculin following stimulation of osteoblast-like cells with ATP and, additionally, assess the formation of vinculin-positive membrane blebs in relation to temporal changes in ATP-induced calcium influx and the magnitude of intracellular traction forces.

## Materials and methods

### Cell culture and transfection

Primary osteoblasts (POBs) were prepared using an outgrowth method as described previously (Curtze et al. 2004). Briefly, bovine metacarpals obtained from the local slaughterhouse were dissected free of soft tissues and rinsed in sterile phosphate-buffered saline. Periosteal samples were collected under sterile conditions and placed in Petri dishes for 3–4 weeks. Cells were cultured in BGJb Medium with Fitton Jackson modification (US Biological) supplemented with 10 % foetal calf serum (Sigma-Aldrich) in a humidified atmosphere of 5 % CO_2_/95 % air at 37 °C. The outgrowing cells reached confluence resulting in a homogeneous monolayer of osteoblast-like cells. Osteogenic lineage was tested immunocytochemically by staining methanol/acetone-fixed cells with antibodies recognizing osteocalcin, procollagen type I, osteopontin, and bone sialoprotein (obtained from Larry W. Fisher, NIH). Positive immunoreactivity was detected by incubating with horseradish-peroxidase-conjugated secondary antibodies in the presence of the enzyme substrate alpha-chloronaphthol, which produced a dark staining of the cells against a white background. Transfection of cells was achieved with a pEGFP expression plasmid coding for human vinculin fused to green-fluorescent protein, termed GFP-vinculin (kindly provided by Tova Volberg, Weizmann Institute, Israel). For controls, a pEGFP-N1 expression vector coding for GFP-tagged STAT1 (signal transducer and activator of transcription 1) was used. Transfection of POBs was performed with the Nanofectin kit (PAA) according to the manufacturer’s recommendations. Twenty-four hours post-transfection, cells were either left untreated or stimulated with ATP (Carl Roth) at a final concentration of 100 μM. Alternatively, GFP-vinculin-expressing cells were treated with 1 μM of bradykinin (Sigma-Aldrich).

### Intracellular [Ca^2+^] measurements

For real-time imaging of intracellular calcium concentrations, POBs grown on glass coverslips were mounted in a silicon chamber and incubated for 60 min at 37 °C with 3 μM Fura-2 fluorescence dye (Molecular Probes) in Ham’s medium (Biochrom) supplemented with 10 mM HEPES (Carl Roth). Coverslips with Fura-2-loaded cells were placed on an inverted Nikon Diaphot IM microscope coupled to a confocal scanner unit. Cells were stimulated by the addition of 100 μM ATP and changes in intracellular [Ca^2+^] concentrations were monitored over at least 100 s. High-pressure twin xenon arc lamps provided alternative excitation at 340 and 380 nm, while emitted light was filtered at 505 nm and collected by a cooled charged-coupled device (CCD) camera (Extended ISIS, Photonic Science), which was controlled by a monochromator (Visitech). The video-signal was digitised and stored in a Quanticell700 image processing system to give a false-colour representation (Applied Imaging-Visitech). The calcium concentration was calculated as the F_340_/F_380_ ratio in n = 15–20 cells. In another set of experiments, the cells were loaded for 60 min with the esterified form of the Ca^2+^-sensitive fluorescent probe Fluo-3/AM (3 μM Fluo-3/AM dissolved in DMSO; Molecular Probe). Recordings were made at room temperature from groups of three to five cells using a 100-W mercury lamp with an excitation wavelength of 488 nm. Fluorescence signals were detected using a narrow band-pass emission filter on a microscope-based spectrofluorometer system. Acquisition of fluorescence data and image analysis was performed using the Quanticell700 software.

### Traction measurement and force calculation

For calculation of traction forces, GFP-vinculin-expressing bovine osteoblasts were cultivated on collagen-coated flexible polyacrylamide sheets at a density of 5,000/cm^2^ containing embedded fluorescent marker beads (Wang and Pelham, 1998). The substrate contained 2 % of a 1:1 mixture of 0.2 and 0.5 μm fluorescent latex micro-beads (Molecular Probes). The mechanical properties of the polyacrylamide gel allow the adhered cells to deform the substratum during their detachment. When the cell is removed, the gel relaxes and the fluorescently labelled marker beads resume their initial position. Fluorescent latex micro-beads could be clearly distinguished from vinculin-rich aggregates due to their spheroidal shape and uniform, small size. The deformations of the substrate were calculated as a matrix of vectors by comparing the fluorescent-light patterns caused by the embedded beads in the presence and absence of the cell (Schwarz et al. 2002; Marganski et al. 2003). Traction force measurements were performed using the LIBTRC software from Dr. Micah Dembo (Boston University, MA, USA) based on a Linux platform.

### Kinetic measurement of individual vinculin clusters

Changes in fluorescence intensities over time related to newly formed GFP-vinculin-rich aggregates were determined in regions of interest (ROIs) located in the cytoplasm. For each ROI, i.e. for each aggregate, an individual fluorescence intensity time course was recorded. Each individual intensity curve was normalized to the maximum intensity and shifted in time in respect to the first moment of the curve in order to calculate an average intensity time course. The mean life time of vinculin clusters in individual ATP-stimulated cells was determined by means of the half-width of the half amplitude method, which was performed separately for the assembly and disassembly phase.

### Statistical analyses

Descriptive statistics were calculated as means ± standard deviations. Numbers of GFP-tagged vinculin clusters were counted at different time points after addition of ATP. Assembly and disassembly times for vinculin clustering were compared using the non-parametric Mann–Whitney-Wilcoxon test. Time-dependent changes in intracellular calcium levels were tested using Friedman repeated measures ANOVA on ranks followed by Tukey tests. Probability values of less than 5 % (*p* < 0.05) were considered statistically significant. All statistical analyses were performed with SigmaStat version 9.0 from Systat Software.

## Results

### Treatment of bovine osteoblasts with ATP or bradykinin induces the formation of vinculin-containing membrane blebs

**Fig. 1:**
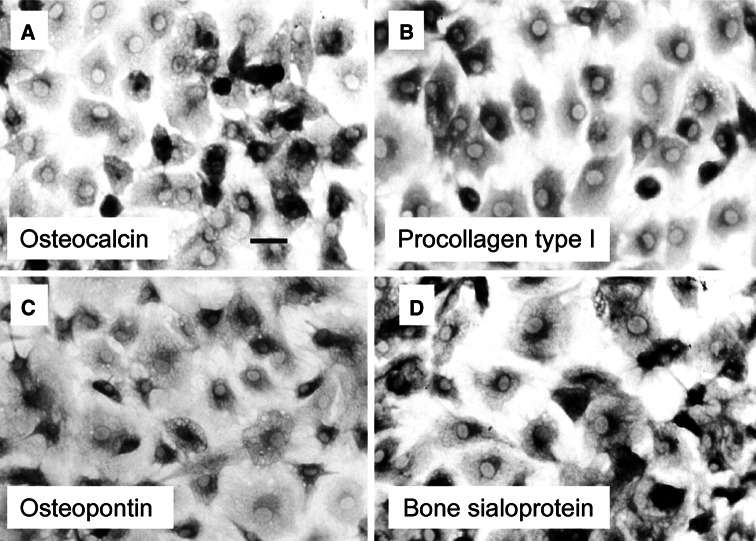
Detection of osteogenic marker proteins in cultured bovine osteoblast-like cells. Shown are immunocytochemical stainings of methanol/acetone-fixed cells for expression of osteocalcin (**a**), procollagen type I (**b**), osteopontin (**c**), and bone sialoprotein (**d**) using horseradish peroxidase-conjugated secondary antibodies. Positive immunoreactivity was visualized by incubating the enzyme with the substrate α-chloronaphthol, which produced a dark staining against a *white* background (*bar* 30 μm)

**Fig. 2:**
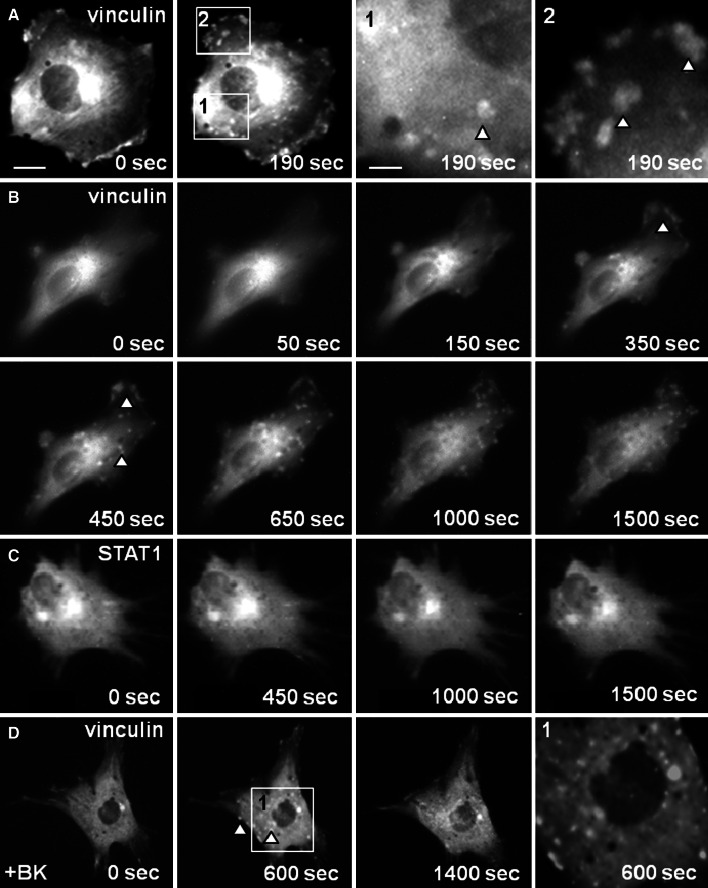
ATP and bradykinin stimulation of bovine primary osteoblasts results in the formation of vinculin-rich membrane clusters. **a** Fluorescence micrographs of a bovine osteoblast expressing recombinant vinculin fused to green-fluorescent protein (GFP) before (0 s) and 190 s after addition of 100 μM ATP. The intracellular distribution of GFP-vinculin before and after ATP treatment at low (*left two images*, *bar* 10 μm) and higher magnification (*right two images*,* bar* 2 μm) is shown. **b** Time-lapse fluorescence microscopy showing de novo formation of vinculin-containing aggregates upon stimulation of cells with ATP (exposure started at t = 0 s). *Arrowheads* mark typical vinculin clusters. **c** No membrane clustering was observed upon ATP treatment in cells expressing GFP-tagged STAT1, used as a control. **d** Transient cluster formation of GFP-vinculin in osteoblasts treated with 1 μM bradykinin (+BK) for 0, 600 and 1,400 s, respectively. The image on the *right* shows vinculin clusters in the perinuclear region at higher magnification

The osteogenic lineage of the cultured cells used in this study, which were obtained by an outgrowth technique, was demonstrated by means of immunocytochemistry, showing the expression of osteocalcin, procollagen type I, osteopontin, and bone sialoprotein (Fig. 1). To monitor the intracellular localization of recombinant vinculin by means of fluorescence microscopy, cells were transfected with an expression plasmid coding for vinculin fused to green-fluorescent protein (GFP). Prior to addition of ATP to the cells, GFP-vinculin was distributed throughout the entire cytoplasm, as determined by a homogeneous fluorescence pattern in the cytosol and the complete absence of fluorescence signals in the nucleus (Fig. 2a). Typically, in resting cells, only a minor fraction of the total intracellular GFP-vinculin pool was incorporated into focal adhesions and located predominantly in a streak-like pattern at the periphery of the cell. Three minutes after addition of ATP to the cells, GFP-tagged vinculin became accumulated in small ephemeral aggregates scattered throughout the plasma, which at higher magnification showed a punctate fluorescence pattern (Fig. 2a).

### Dynamic changes in vinculin localization resulting from ATP stimulation of cells

In order to monitor the time course of vinculin redistribution in living osteoblasts before and after treatment with 100 μM of ATP, time-lapse fluorescence microscopy of GFP-vinculin-expressing cells was performed (Fig. 2b). After a lag phase of approximately 100 s following exposure to ATP, fluorescently labelled vinculin started to accumulate in lamellipodial protrusions at the rim of the cells as well as in numerous small, dot-like aggregates scattered throughout the cytoplasm. The numbers of these GFP-vinculin-rich membrane structures increased over time up to 650 s after addition of the nucleotide and then slowly decreased to pre-stimulation counts. Occasionally, vinculin-containing aggregates located at the periphery conflated to form larger protrusions and were transformed into lamellipodial extensions. These dynamic changes in vinculin distribution were fully reversible and we found no evidence for apoptotic cell death in the treated cells even after long observation periods. In control experiments using GFP-tagged signal transducer and activator of transcription 1 (STAT1) as a marker protein, treatment with ATP at the same concentration did not result in an altered intracellular localization, demonstrating the specificity of the dynamic vinculin redistribution (Fig. 2c). However, when GFP-vinculin-expressing cells were treated with 1 μM bradykinin instead of ATP, a similar kinetics of transient vinculin clustering was observed (Fig. 2d).

### Half-life of vinculin-containing focal membrane structures

**Fig. 3:**
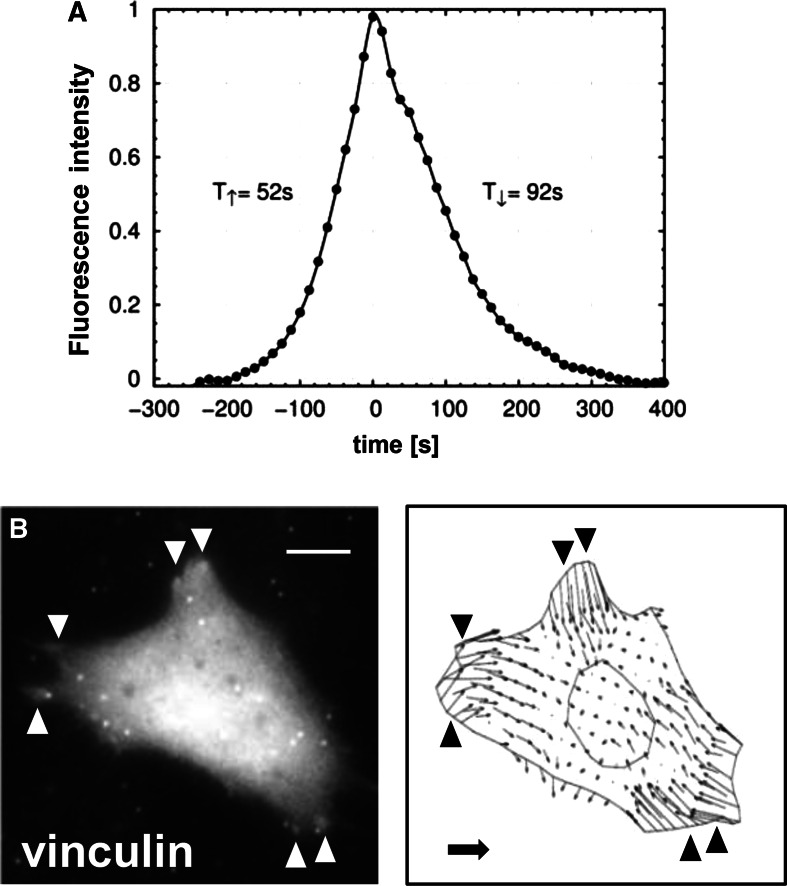
**a** Kinetics of the assembly and disassembly of single GFP-vinculin-containing membrane clusters. Changes in fluorescence intensity were averaged over several aggregate-containing ROIs in the cytoplasm and plotted over time. The peak of fluorescence intensity was set to t = 0 s. **b** Vinculin-rich aggregates co-localize at sites of high traction force magnitude. Intracellular traction forces were measured by substrate deformation using fluorescent latex micro-beads embedded in a collagen-coated flexible polyacrylamide sheet. The fluorescent micro-beads scattered throughout the substratum were clearly distinguishable from the larger and more weakly labelled vinculin clusters. The images depict a fluorescence micrograph of a GFP-vinculin-expressing osteoblast (*left image*) exhibiting dot-like aggregates at its cell periphery (marked with *arrowheads*, *bar* 10 µm) and the corresponding vector map of traction forces within the same cell (*right image)*. The scale vector represents 500 N/m^2^ (total force of 4,500 μN/cell)

Given the ephemeral nature of the ATP-induced membrane excrescences, we next determined the formation kinetics of individual vinculin-rich aggregates using the half-width of the half amplitude method, which was performed separately for the assembly and disassembly phase. The results of these measurements demonstrated a mean assembly time of 66.7 ± 13.3 s (Fig. 3a). Compared to this rapid formation, the subsequent disassembly time was slightly longer (99.0 ± 6.6 s), and there was virtually no stationary phase at maximal fluorescence intensity.

### Vinculin-rich aggregates localize to areas of high traction forces

We next determined traction forces in GFP-vinculin-expressing osteoblasts and related the magnitudes of the differential traction stress at each pixel in the cell area to the occurrence of stress-induced vinculin aggregates. For this purpose, traction-force vector maps were calculated from individual cells cultivated on collagen-coated flexible polyacrylamide sheets, which contained fluorescent marker beads of uniform size and spheroid shape (Curtze et al. 2004). The spatial distribution of traction forces was analysed by comparing the fluorescence light patterns obtained from embedded beads in the presence or absence of cells. Substrate deformation traction forces were quantified by the displacement of the fluorescent marker beads embedded in the gel and resulted in a matrix of vectors within the cell. These experiments showed that most of the prominent deformations were found at the edge of the cell, as expected, and, more importantly, localized to areas with a high probability of assembly of vinculin-rich aggregates, as judged from their frequent occurrence at these sites (Fig. 3b).

### Time course of calcium response in relation to vinculin clustering

**Fig. 4:**
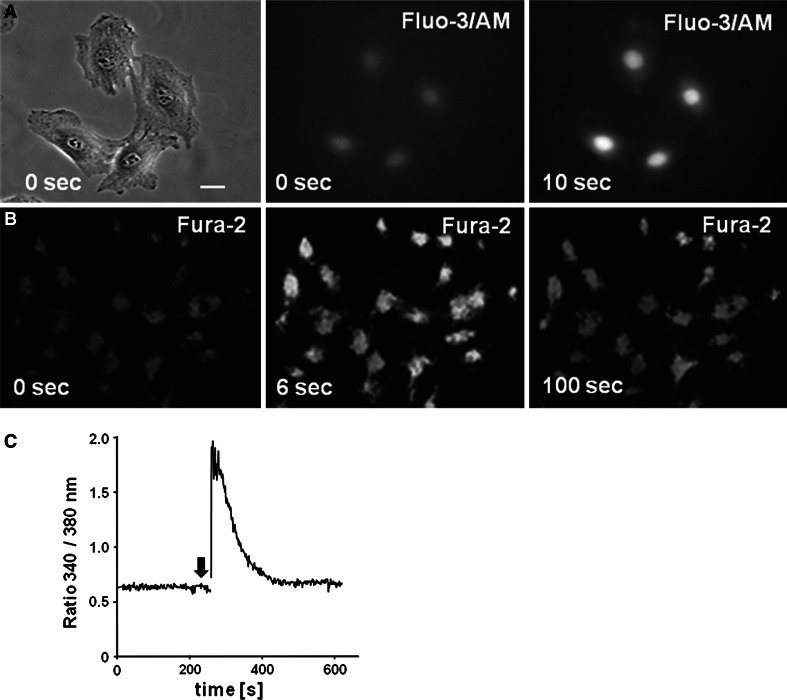
Kinetics of calcium influx in bovine osteoblasts stimulated with high concentrations of ATP. **a** Cultured cells were loaded with Fluo-3/AM and subsequently treated with 100 μM of the nucleotide. Exposure to ATP resulted in an increase in Fluo-3/AM fluorescence intensity. A phase contrast microscopical image (*left*,* bar* 8 μm) and the corresponding fluorescence staining before (*middle*, 0 sec) and after addition of ATP (*right*, 10 sec) are shown. **b** Fura-2AM fluorescence signal intensities emitted at 505 nm were measured before (0 s), and 6 and 100 s after ATP treatment using excitation wavelengths of 340 and 380 nm, respectively. **c** Changes in the ratio F_340_/F_380_ were calculated from a typical cell before and after stimulation with ATP (marked with an *arrow*)

In order to assess the time course of vinculin clustering in relation to the ligand-induced calcium transients observed, we determined intracellular calcium levels in isolated bovine osteoblasts in response to ATP stimuli. Calcium concentrations were measured before and after stimulation of cells with 100 μM ATP by means of Fluo-3AM imaging (Fig. 4a). Results showed significant [Ca^2+^]_i_ increments during the stimulation phase as compared to pre-stimulation baseline. Serial images using Fura-2 confirmed the rapid ATP-induced calcium rise, which peaked already within the first 30 s following addition of purinergic agonist to the cells (Fig. 4b, c). As expected, no calcium response was detected in cells exposed to Ham′s solution alone (data not shown).

## Discussion

Based on fluorescence studies, we have previously shown that ATP treatment of osteoblast-like cells results in the transient accumulation of paxillin in membrane blebs (Silber et al. 2012). In the present study, we have extended this observation on changes in the cortical cytoskeleton by focussing on the intracellular redistribution of vinculin. In particular, we have assessed whether the focal adhesion protein vinculin is also subjected to an intracellular aggregation upon stimulation of cells with high concentrations of ATP. A transient clustering of the focal adhesion protein vinculin was observed starting approximately 100 s after applying the ATP stimulus to the cells. Time-lapse fluorescence microscopy of ATP-treated, GFP-vinculin-expressing cells demonstrated a punctate localization pattern in the cytosol, which clearly differed from the streak-like appearance of vinculin in substratum-attached focal adhesions. The ephemeral nature of the vinculin-rich aggregates became evident when we determined their short half-life by video-tracking microscopy and found that it did not exceed 200 s. Several generations of locally restricted vinculin clusters were formed and consecutively disrupted during the course of the transient morphological alterations that accompanied binding of the purinergic agonist to P2 receptors. Generally, only a minor fraction of the total GFP-tagged vinculin was sequestrated into these transient structures, while the majority of vinculin molecules remained homogeneously distributed throughout the cytoplasm, suggesting that there was a dynamic exchange between the two pools of structurally bound and non-bound vinculin (Wolfenson et al. 2009; Tan et al. 2010).

Furthermore, our data demonstrate that the ATP-induced increase in [Ca^2+^]_i_ clearly precedes the membrane clustering of vinculin, suggesting that intracellular calcium transients trigger a cascade of signalling events which ultimately results in the incorporation of vinculin in membrane-associated focal aggregates, rather than being the direct cause of vinculin clustering. Nevertheless, the release of intracellular free calcium and the subsequent sequestration of vinculin molecules at membrane protuberances are both fast processes, the latter accompanying the ATP-induced morphological changes of the cells. The reversible clustering of vinculin in ATP-stimulated osteoblasts reported here closely resembles the dynamic exchange of paxillin observed under the same stimulation condition, thus confirming the pivotal role of the cortical cytoskeleton for the execution of purinergic signal transduction (Silber et al. 2012).

The molecular mechanisms underlying agonist-induced membrane blebbing are still largely unknown. Interestingly, Panupinthu and colleagues have shown that inhibition of Rho-associated kinase (ROCK) abolishes P2X_7_-induced membrane blebbing in calvarial osteoblasts (Panupinthu et al. 2007). Their observation that membrane blebbing in osteoblasts is sensitive to the selective ROCK inhibitor Y-27632 suggests that assembly and contraction of the cortical actomyosin filament system cause the formation of transient membrane blebs. Based on their observation that blockade of lysophosphatidic acid (LPA) receptors suppressed blebbing in response to nucleotide stimulation without affecting P2X_7_-induced pore formation, the authors proposed a signal pathway that links purinergic receptor stimulation through phospholipases to the production of LPA and activation of ROCK.

Our finding that vinculin is involved in the reorganization of the cortical cytoskeleton following ATP stimulation suggests that this protein is more than just a simple linker or adaptor molecule that couples components of the cadherin and integrin family of cell adhesion molecules to the actin filament network (Humphries et al. 2007; Wolfenson et al. 2011). The traction force measurements provided evidence that newly formed vinculin clusters are engaged in cell spreading and modulate membrane ruffling, since we noted a high probability for the assembly of these clusters at sites of high intracellular tension force. From these data, it is conceivable that vinculin functions to coordinate membrane blebbing and suppression of cell migration by stabilizing cell–cell and cell–matrix contacts of stimulated cells in their normal tissue microenvironment.

In summary, our data show that ATP stimulation of osteoblasts triggers the rapid and reversible accumulation of vinculin in multiple, dot-like aggregates, which accompany the dynamic changes in cell morphology induced by this extracellular nucleotide. When vinculin is engaged in membrane herniation and de novo formation of lamellipodial protrusions, the ligand-induced increase in intracellular calcium concentrations has already been accomplished and calcium levels have returned to the resting state. Traction force measurements in untreated osteoblasts demonstrate that vinculin clusters are localized predominantly in regions of high traction-force magnitude, thus confirming that this focal-adhesion protein plays an important role in the cytoskeletal rearrangements observed in mechanically and/or chemically stimulated osteoblast-derived cells.
